# Conservation Analysis of B-Cell Allergen Epitopes to Predict Clinical Cross-Reactivity Between Shellfish and Inhalant Invertebrate Allergens

**DOI:** 10.3389/fimmu.2019.02676

**Published:** 2019-11-19

**Authors:** Roni Nugraha, Sandip D. Kamath, Elecia Johnston, Shaymaviswanathan Karnaneedi, Thimo Ruethers, Andreas L. Lopata

**Affiliations:** ^1^Australian Institute of Tropical Health and Medicine, James Cook University, Townsville, QLD, Australia; ^2^Department of Molecular and Cell Biology, College of Public Health, Medical and Veterinary Science, James Cook University, Townsville, QLD, Australia; ^3^Department of Aquatic Product Technology, Bogor Agricultural University, Bogor, Indonesia; ^4^Centre for Food and Allergy Research, Murdoch Children's Research Institute, Melbourne, VIC, Australia

**Keywords:** shellfish allergens, IgE epitopes, B-cell epitopes, shellfish cross-reactivity, tropomyosin

## Abstract

Understanding and predicting an individual's clinical cross-reactivity to related allergens is a key to better management, treatment and progression of novel therapeutics for food allergy. In food allergy, clinical cross-reactivity is observed in patients reacting to unexpected allergen sources containing the same allergenic protein or antibody binding patches (epitopes), often resulting in severe allergic reactions. Shellfish allergy affects up to 2% of the world population and persists for life in most patients. The diagnosis of shellfish allergy is however often challenging due to reported clinical cross-reactivity to other invertebrates including mites and cockroaches. Prediction of cross-reactivity can be achieved utilizing an in-depth analysis of a few selected IgE-antibody binding epitopes. We combined available experimentally proven IgE-binding epitopes with informatics-based cross-reactivity prediction modeling to assist in the identification of clinical cross-reactive biomarkers on shellfish allergens. This knowledge can be translated into prevention and treatment of allergic diseases. To overcome the problem of predicting IgE cross-reactivity of shellfish allergens we developed an epitope conservation model using IgE binding epitopes available in the Immune Epitope Database and Analysis Resource (http://www.iedb.org/). We applied this method to a set of four different shrimp allergens, and successfully identified several non-cross-reactive as well as cross-reactive epitopes, which have been experimentally established to cross-react. Based on these findings we suggest that this method can be used for advanced component-resolved-diagnosis to identify patients sensitized to a specific shellfish group and distinguish from patients with extensive cross-reactivity to ingested and inhaled allergens from invertebrate sources.

## Introduction

The prevalence of food allergy is steadily increasing over the past decade, with ~4% of adults and up to 10% of children having some type of food allergy. The shellfish group is included among the “Big Eight” food groups that are responsible for more than 90% of all food allergy cases (1). It is estimated that 2% of the general population are affected by food allergy to shellfish (2). Shellfish allergy has, similar to peanut allergy, one of the highest rates of food-induced anaphylaxis with nearly 42% among affected adults and 12% in children (3). Shellfish allergy is typically persistent, with only 13% of patients likely to outgrow their shellfish allergy (4).

In the sensitized individual, subsequent exposure to the shellfish allergen, via ingestion or inhalation, triggers the generation of specific IgE antibodies by activated B-cells of the immune system. These antibodies subsequently bind to immune effector cells, including mast cells and basophils, resulting in degranulation and clinical manifestation of allergic symptoms (5). Currently, eight proteins from different shellfish species are known as the main provocateur of shellfish allergy and have been registered in the World Health Organization and International Union of Immunological Societies (WHO/IUIS) Allergen Nomenclature Sub-committee (2).

Two allergens of major importance in the development of shellfish allergy are the muscle protein tropomyosin (TM) and the enzyme arginine kinase (AK). Tropomyosin is the major allergen of shellfish allergy with specific IgE antibody responses in 60–80% of shellfish allergic patients recognizing this protein, and it is suggested to be a good biomarker of severe clinical-reactivity to shellfish (6, 7). TM has been identified as a major allergen in over 14 crustacean and 5 mollusc species (www.allergen.org). Meanwhile, AK has been considered as the second invertebrate pan-allergen, implicating allergy cross-reactivity to shellfish, mite and insect. IgE-sensitization toward AK has been found in 10–51% of shrimp allergic patients (2).

The primary structure of TM's alpha-helical coiled-coil dimeric protein is highly conserved across various invertebrates and has been identified in over 150 species, including insects and mites (8). This structural similarity seems to be one of the main reason for the high degree of immunological cross-reactivity and as a result, is responsible for inducing allergic immune responses in over 30% of the world-population (9, 10). Furthermore, the immunogenic properties of TM have made it a prominent vaccine candidate for several pathogens in livestock, including nematodes, filaria, scabies and ticks (5–7).

Edible crustacean and mollusc are commonly discussed as “shellfish.” However, the group of “shellfish” comprises the two invertebrate phyla arthropods and molluscs. Although all shellfish are invertebrate animals, these two groups are very distinct in evolutionary terms and subsequently contain different molecular repertoires of food allergens. In fact, crustacean are placed closer to insects and arachnids (spiders), and this seems to be the major factor for molecular sensitization and clinical reactivity between crustacean, dust-mite, insects and parasites (11). Severe acute allergic reactions upon accidental ingestion of different shellfish species and insects have been observed for a long time and studies attempted to understand the underlying immunological cross-reactivity have been conducted (12, 13). Immunological cross-reactivity was also demonstrated in unexpected populations, never exposed to shrimp TM (14). Tropomyosin from shrimp and mite seem to cross-react considerably, as recently demonstrated in patients clinically reacting to TM in ingested insects (15, 16). Furthermore, IgE responses to TM in mite and the parasite Ascaris are significant risk factors for asthma (17). While the clinical relevance of cross-reactivity to TM is known for as long time, lack of specific diagnostic tools are the main problem for correct diagnosis of crustacean and/or mollusc allergy and extensive immunological cross-reactivity with other invertebrate allergen sources containing similar proteins.

Cross-reactivity occurs when the IgE antibodies recognize identical or very similar protein patches (epitopes) from different proteins as compared to the primary sensitizing protein (18). IgE cross-reactivity to unrelated peanut allergens has recently been demonstrated (19), resulting from amino acid similarities of short peptides. Furthermore, it is known that phylogenetically related species often have similar proteins, and this similarity can implicate IgE cross-reactivity (20). Allergens which share highly conserved protein sequences, but also structure and function, can be termed pan-allergens if they are responsible for antibody binding cross-reactivity and subsequent clinical cross-reactivity (21).

Cross-reactivity of related proteins could be predicted computationally by comparing the identity of the amino acid sequence to the known allergen. Aalberse (22) reviewed potentially cross-reactive structures of known allergens and noted that proteins with >50% identity throughout the length of the protein compared to an allergen are likely cross-reactive. However, some recent studies (19, 23, 24) demonstrated that this predictive value is only useful to predict the allergenicity of new protein of unknown structure but is not accurate to predict cross-reactivity. This is of particular importance for invertebrate tropomyosin, which shares over 50% of amino acid identify with human tropomyosin. Moreover, in many cases the demonstrated cross-reactivity is patient-specific and seem only to occur when the patient IgE antibodies bind to conserved epitopes. In the context of food allergy, sequential IgE binding epitopes seem to be much more relevant as conformational epitopes are easily degraded due to the digestion in the gastrointestinal tract (25).

Based on these observations, we consider that a large-scale analysis of sequential IgE epitope conservation is of great importance for predicting clinical cross-reactivity between crustacean and mollusc, as well as mite and cockroach, in allergic patients. While there are eight allergenic proteins known among different shellfish (26), conclusive epitope data are only available for shrimp allergens including TM, AK, SCP, and MLC. We developed a database of positive IgE-binding epitopes of these shrimp allergens collected from the Immune Epitope Database (IEDB). Subsequently, we determined the conserved epitope sequences responsible for cross-reactivity using the Epitope Conservancy Analysis program (27). Shrimp allergen epitopes were considered to be conserved if the sequence of the other invertebrate homologous peptide had less than two amino acid mismatches (28–31). These epitopes could be used to design better predictive diagnostic tools for shellfish allergic patients as well as enabling the early detection of risk factors for developing food allergy to shellfish in dust mite allergic patients, thereby leading to the improved management of this severe clinical condition.

## Methods

### Collection of Shrimp Allergens IgE-Binding Epitopes

The dataset for the subsequent analysis was built from available IgE binding epitopes from four shrimp allergens: tropomyosin (TM), arginine kinase (AK), myosin light chain (MLC), and sarcoplasmic calcium-binding protein (SCBP). The data were assembled from the Immune Epitope Database and Analysis Resource (IEDB) (32). The collected epitopes were restricted to peptides with positive serum IgE antibody binding from patients with confirmed shrimp allergy.

### Sequence Retrieval and Phylogenetic Analysis

Protein sequences were obtained from the UniProt database and aligned using MUSCLE v3.8.31 (33). Different numbers of protein sequences could be retrieved for TM, AK, MLC, and SCBP, with, respectively 54, 30, 20, and 10 protein sequences. The selected sequences represent 18 different crustacean and 30 mollusc species within the shellfish group. The subsequent phylogenetic analyses of relatedness of proteins were performed using MrBayes v3.2.6 (34) (50,000 generations) and RAxML v8.2.9 (35) (1,000 replicates) via the CIPRES Science Gateway (36).

### Conservation Analysis of Shrimp Allergens in Invertebrate Species

The conservation of amino acid residues for each allergen among the different invertebrate species was estimated using the Rate4Site algorithm in Consurf (37) server by calculating position-specific evolutionary rates under an empirical Bayesian methodology. The rates were normalized and grouped into 9 grades, where high conserved residues receive a score of 9 and very variable residues receive a score of 1. The conservation rate of the amino acids was then mapped using Chimera (38) to the structure model of the allergens generated by Swiss-Model (39).

### Conservation Analysis of IgE-Binding Epitopes of Shrimp Allergens

The degree of conservation of the epitopes within the sequences of the respective allergens was calculated using conservancy analysis tool (27) on the IEDB website. The degree of conservation of an epitope is calculated as the fraction of the protein sequence that matched the aligned epitope above a chosen identity level. An epitope was considered to be conserved if the homologous peptide had less than two amino acid mismatches.

### Data Analysis

Data analysis was performed using GraphPad Prism version 7. One-way ANOVA was applied to determine the statistically significant difference of the conserved epitopes between groups of invertebrate species.

## Results

### Amino Acid Sequence Analysis of Shrimp Allergens

**Figure 1:**
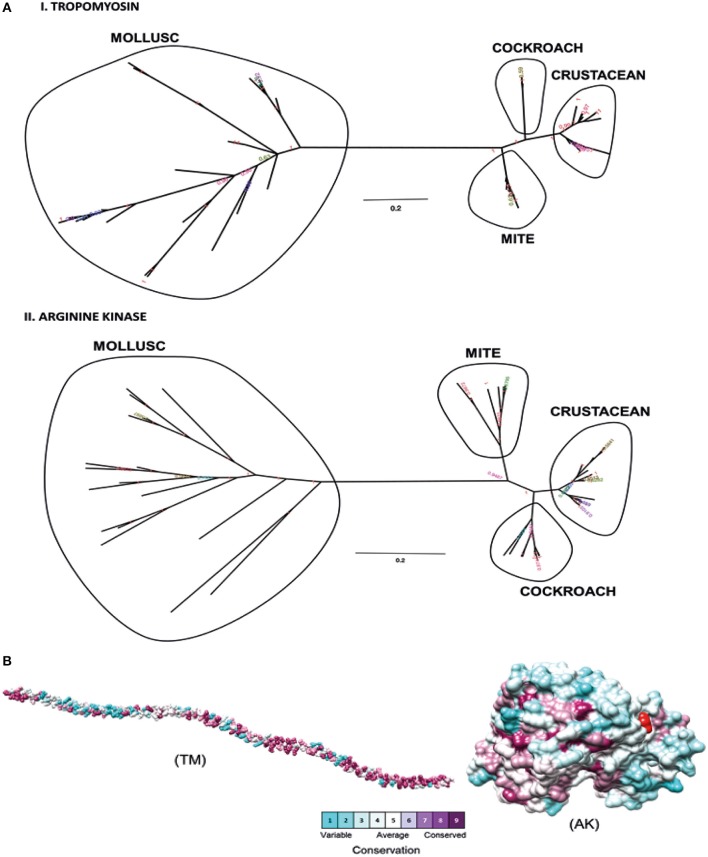
Molecular evolutionary analysis of shellfish allergens. **(A)** The phylogenetic tree was drawn using Bayesian and Maximum Likelihood approach for (I) Tropomyosin and (II) Arginine kinase. The tree is drawn to scale, with branch lengths in the same units as those of the evolutionary distances used to infer the phylogenetic tree. **(B)** Conservation analysis of individual amino acids in tropomyosin (TM) and arginine kinase (AK) was determined using the Consurf server. The conservation grades were mapped onto the query sequence and structure using the ConSurf color-code, with cyan-through-purple corresponding to a variable (grade 1)-through-conserved (grade 9) positions.

Phylogenetic analysis was performed to determine the relationships between shrimp allergen sequences in different invertebrate groups and to infer the evolutionary trends among the wide representation of the allergens. In this study, four shrimp allergens, TM, AK, SCP, and MLC were selected based on the availability of the IgE-binding epitopes. A dataset of TM, AK, SCP, and MLC protein sequences from crustaceans and molluscs species in UniProt database was assembled to construct a tree using the Maximum Likelihood and Bayesian approach. A consensus tree generated for all protein groups showed similar topologies with good branch support (>70%) for major branches for TM and AK (Figure 1A). These trees, particularly for TM, are in good agreement with previously published trees (40, 41), demonstrating the expected distant phylogenetic relationship between crustacean and mollusc. Crustacean clustered closer with other allergy-causing arthropods, including mite and cockroach, while the mollusc form a distinct cluster.

The degree of evolutionary conservation at individual amino acid sites of TM and AK were determined using Consurf server by applying the Rate4Site algorithm. In ConSurf, the evolutionary rate is estimated based on the evolutionary relatedness between the protein and its homologs and considers the similarity between amino acids as reflected in the substitutions matrix. The conservation grades identified using ConSurf are mapped to the query sequence and/or structure using the ConSurf color-code, with cyan-through-purple corresponding to a variable (grade 1)-through-conserved (grade 9) positions (Figure 1B). As the analysis can only be conducted if there are at least five homologous proteins, the conservation analysis could only be conducted for the two major allergens, TM and AK, but not for SCP and MLC. In general, TM had more conserved amino acids than AK. Most of the conserved amino acids in the TM were located at the N- and C-terminal regions. A total of 175 out of 284 TM residues had conservation grades >5, while 86 residues (30%) had the full grade 9. For AK, 200 out of 356 residues had conservation grades >5, of which 96 residues (27%) had grade 9.

### Conservation of IgE Binding Epitopes of Shrimp Allergens

**Figure 2:**
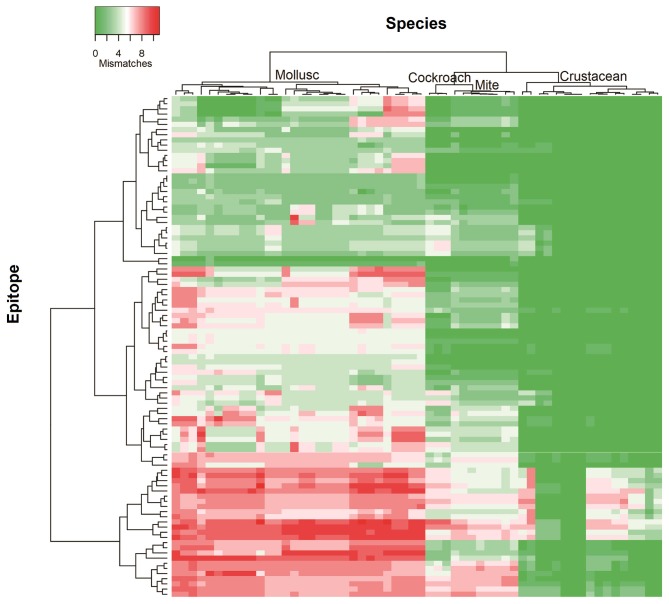
Heatmap representing number of mismatches in homologous peptide of shrimp TM IgE-binding epitopes in different invertebrate species. The heatmap was generated using Heatmapper and clustered using Manhattan distance metric approach. The colors grading as indicated in the top left represents the number of amino acid mismatches found in the homologous peptides of epitopes. Green indicates no mismatches and red indicates maximum mismatches.

To determine the likelihood of cross-reactivity between shrimp and other invertebrates, the conservation analysis of epitopes in those groups was conducted. A database of linear 176 epitopes was generated, containing 96 epitopes from TM, 39 epitopes from AK, 27 epitopes from MLC, and 12 epitopes from SCBP. These epitopes were identified to be recognized by IgE antibodies from over 100 patients with shellfish allergy as determined in previous studies (6, 29, 42–45). The epitope conservation was determined using epitope conservancy analysis tool within the IEDB webpage. Epitopes were conserved when they share less than two amino acid mismatches within the aligned sequence. The conservation analysis showed a similar trend for TM and AK (Figure 2). The number of species containing peptide regions similar with the epitopes was found to be the highest within the crustacean followed by cockroach, mite, and mollusc.

**Figure 3:**
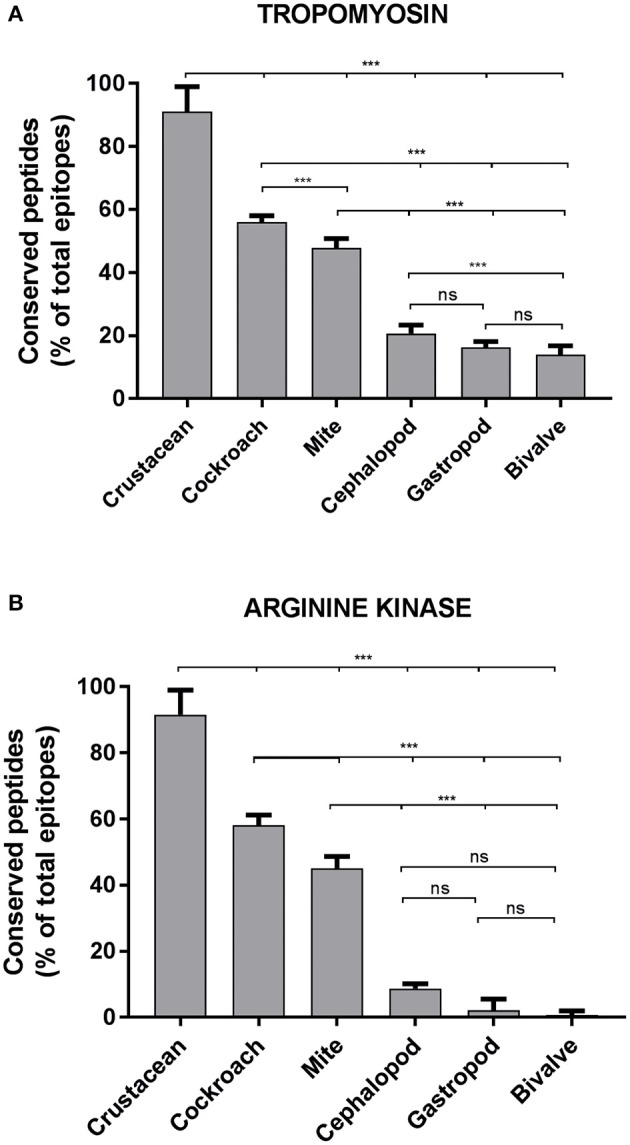
Percentage of conserved shrimp IgE-binding epitopes between invertebrate species. Ninety-Eight B-cell epitopes from tropomyosin **(A)** and 39 B-cell epitopes from arginine kinase **(B)** were examined for their conservation within shellfish and between other allergenic invertebrate species. An epitope was considered conserved if the sequence matched to a homolog or a peptide variant with not more than 2 amino acid substitutions in another species. Significance differences (*p* < 0.05) were calculated using One-way ANOVA. ns, not significant, ^***^p < 0.05.

Approximately, 91% of the IgE epitopes were found to be conserved in crustacean, 56% in cockroach and 48% in mite species. The number of conserved epitopes in crustacean, cockroach, and mites epitopes were significantly higher than in the three mollusc groups where only <20% of TM epitopes were conserved (Figure 3A) and even less for AK with 9% of the epitopes (Figure 3B). Nevertheless, these conservations were very high with the majority having only one or two amino acid mismatches. Within the molluscs, the cephalopods had the highest number of conserved epitopes, followed by gastropods and bivalves. The analysis of 20 MLC and 10 SCBP proteins resulted in no conserved epitopes to be identified within the mollusc group.

### Invertebrate Allergen Pan-Epitopes

**Figure 4:**
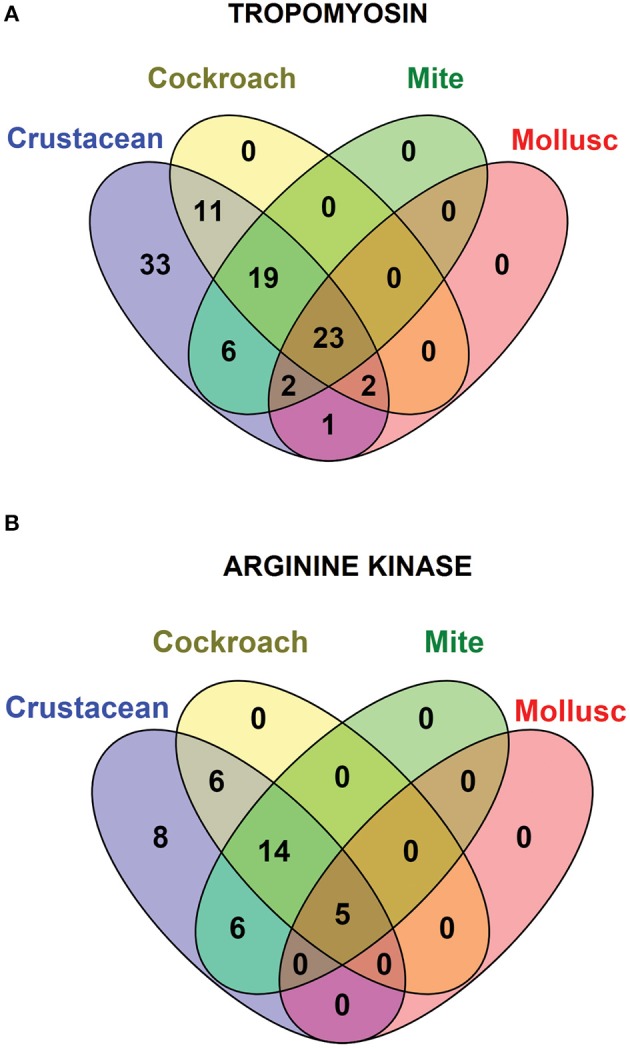
Venn diagram showing the similarities among the conserved shrimp IgE-binding epitopes across invertebrate species. In total 96 epitopes on TM **(A)** and 39 on AK **(B)** were analyzed.

Species-specific conservation analysis of IgE binding epitopes was carried out to identify the allergen epitope sequences that could be termed “pan-epitope.” Of the 97 shrimp TM IgE-binding epitopes, 22 invertebrate pan-epitope were identified (Figure 4A). These epitopes were conserved and shared by crustacean, cockroach, mite as well as the mollusc. The epitope sequences are summarized in Table 1. The species belonging to the Arthropoda—crustacean, cockroach, and mite, shared 19 IgE-binding epitopes, while crustacean-cockroach and crustacean-mite shared 11 and 6 epitopes, respectively. Thirty-three IgE-binding epitopes were specific to crustaceans and may be used to diagnose crustacean-specific IgE sensitization.

**Table 1:** Sequences of TM IgE-binding epitopes responsible for cross-reactivity between crustacean, cockroach, mite, and mollusc in prawn allergic patients and their presentation in each invertebrate group.

**Epitope sequence**	**Position**	**Presentation (%)**			
		**Crustacean (%)**	**Cockroach (%)**	**Mite (%)**	**Mollusc (%)**
MDAIKKKMQAMKLEK	1–15	100	100	100	77
IKKKMQAMKLEKDNA	4–18	100	100	100	10
VAALNRRIQLLEEDL	85–99	100	100	100	3
LNRRIQLLEEDLERS	88–102	100	100	100	33
NRRIQLLEEDLERSEER	89–105	100	100	100	33
RIQLLEEDLER	91–101	100	100	100	43
RIQLLEEDLERSEER	91–105	100	100	100	33
EASQAADESERMRK	115–128	100	100	100	50
EASQAADESERMRKV	115–129	100	100	78	50
LENQLKEA	144–151	100	100	100	37
LAEEADRKYDEVARK	154–168	100	100	100	10
EADRKYDEVARKLAM	157–171	100	100	100	10
ESKIVELEEELRVVG	187–201	100	100	100	17
IVELEEELRVVGNNL	190–204	100	100	100	20
LEEELRVVGNNLKSL	193–207	100	100	100	50
KEVDRLEDELVNEKEKYKSI	241–260	100	100	100	60
ERSVQKLQKEVDRLEDE	243–259	100	100	100	90
QKLQKEVDRLEDELV	247–261	100	100	100	93
LQKEVDRLEDELV	249–261	100	100	100	100
QKEVDRLEDELVNEK	250–264	100	100	100	93
KEVDRLEDE	251–259	100	100	100	100
VDRLEDELVNEKEKY	253–267	100	100	100	63

In contrast, only 5 of the 39 shrimp AK IgE-binding epitopes were conserved across crustacean, cockroach, mite, and mollusc (Figure 4B and Table 2) and unlike TM IgE-binding epitopes, only few AK IgE-binding epitopes were specific to crustacean. Table 1 shows that most of the invertebrate TM pan-epitopes are located at the N- and C-terminals of the TM sequence. The epitopes at the amino acid position 243–264 were of importance for the cross-reactivity across the tested sequences due to high conservation in over 90% of the invertebrate species analyzed.

**Table 2:** Sequences of AK IgE-binding epitopes responsible for cross-reactivity between crustacean, cockroach, mite, and mollusc in prawn allergic patients and their presentation in each invertebrate group.

**Epitope sequence**	**Position**	**Present in (%)**			
		**Crustacean (%)**	**Cockroach (%)**	**Mite (%)**	**Mollusc (%)**
SLLKKYLTKEVFDKL	25–39	57	100	33	9
EGGIYDISNKRRMGL	319–333	100	100	67	36
IYDISNKRRMGLTEF	322–336	100	100	67	55
ISNKRRMGLTEFQAV	325–339	100	100	100	45
KRRMGLTEFQAVKEM	328–342	100	50	100	27

### Shellfish Allergen Pan-Epitopes

Avoidance of other shellfish species including molluscs is one of the management strategies for shrimp-allergic patients. However, Figure 1 shows that the major allergenic proteins from shrimps and mollusc are distinctly different, supported by previous studies showing that cross-reactivity between shrimp and mollusc is species-specific (46). Based on this rationale, further detailed analysis of shrimp IgE-binding epitope conservation across three edible mollusc classes, including bivalves, cephalopods and gastropods were carried out. In total, 28 shrimp TM and 5 AK IgE-binding epitope sequences were aligned with less than two amino acid mismatches with at least one mollusc species (Figure 4).

**Figure 5:**
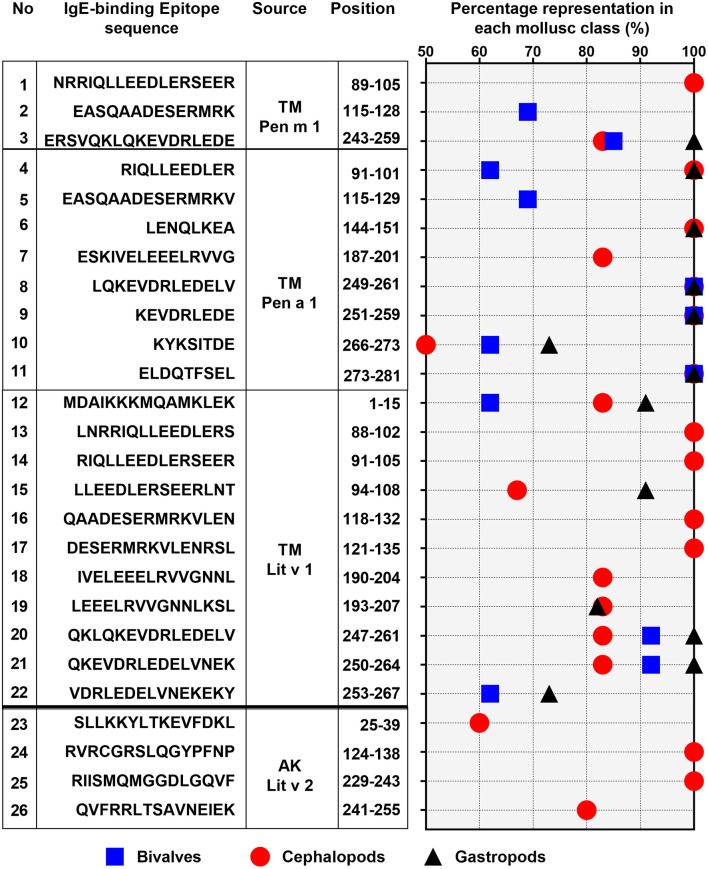
Percentage representation of conserved epitopes in the three mollusc classes: bivalves (square), cephalopod (circle), and gastropod (triangle). Only epitopes which are present in over 50% of each mollusc classes are shown. The epitope allergen sources, their amino acid sequences and positions in the protein are indicated on the left.

Of the 28 conserved TM epitopes, only 22 epitopes were present in over 50% of each mollusc classes, with three epitopes were conserved in all of the mollusc species (Figure 5). Detailed analysis of the conserved epitopes revealed that half of the amino acid residues in shrimp TM were responsible for cross-reactivity to at least one species of mollusc and these amino acids were distributed across the entire protein sequence. Some of the epitopes showed group-specific conservation, such as Epitope 3, 14, 15, 17, and 18 in Figure 5. Unlike TM, where the conserved epitopes were aligned with various species of mollusc, the conserved epitopes of AK were mostly aligned with peptides of the cephalopod. Of the five conserved AK epitopes, only four epitopes were present in over 50% of mollusc species, with two of those were aligned to all species of cephalopods (Figure 5). While the complete amino acid sequence of the 4 analyzed shrimp TMs are identical, the length and composition of the identified IgE epitopes differs. Nevertheless, the overall trend of high conservation to cephalopod peptides is similar, as well as the low conservation to bivalves.

**Figure 6:**
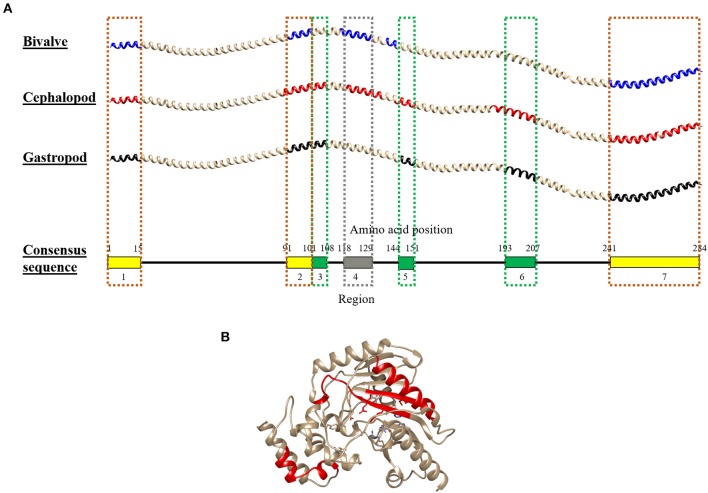
Molecular modeling of the conserved IgE-binding epitopes of **(A)** tropomyosin (TM) and **(B)** arginine kinase (AK) that are presented in over 50% of each mollusc class. For tropomyosin, the epitopes were remapped to their consensus tropomyosin sequence and color-coded based on mollusc classes in which the conserved epitopes are found: yellow (all mollusc classes), green (cephalopod and gastropod), and gray (bivalve and cephalopod). The red color in AK protein model highlights the conserved sequence. The protein structure of TM and AK were modeled using SWISS-MODEL based on reference proteins 1C1G and 4BG4, respectively.

Combining the conserved epitopes divided the shrimp TM into three possible cross-reactivity scenarios, located on distinct areas on the TM allergen. Three regions with a total of 35 amino acids residues, or 12%, of the total 284 amino acids were conserved across all classes of the mollusc phyla (Figure 6, yellow boxes) and responsible for shellfish pan-allergy. Three regions (Figure 6, green boxes) were conserved within the cephalopods and gastropods, and one region (Figure 6, gray box) was conserved within the bivalves and cephalopods.

### Decision Tree to Identify Potential Crustacean-Mollusc Cross-Reactivity in Shrimp-Allergic Patients

**Figure 7:**
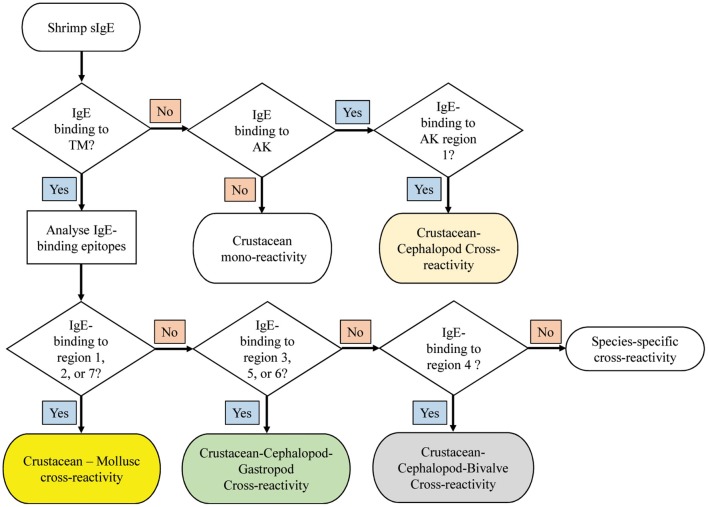
Decision tree to diagnose potential molecular cross-reactivity to the invertebrate allergens TM and AK in shrimp allergic patients based on the reactivity to specific IgE epitopes. The region numbers refer to the epitope mapping in Figure 5.

A decision tree was developed to identify potential shrimp-mollusc cross-reactivity in shrimp-allergic patients based on the conservation patterns of shrimp TM and AK IgE-binding epitopes in mollusc species (Figure 7). In the diagnosis of shrimp allergy, where clinical history does not give a clear conclusion, sensitization tests against a whole shrimp extract and specific allergens are needed prior to oral food challenge. If TM-specific IgE results are positive, with quantitative IgE-levels to TM being similar than to the whole protein extract, immune-dominant sensitization to shellfish TM is likely, and broad (serological) cross-reactivity to other shellfish species is to be expected. Analysing IgE-binding to shrimp TM epitopes can further improve the diagnosis of cross-reactivity. Four reactivity patterns are suggested including; crustacean mono-reactivity, crustacean-mollusc cross-reactivity, crustacean-cephalopod-gastropod cross-reactivity, and crustacean-cephalopod-bivalve cross-reactivity. However, if TM-specific IgE are not present (negative), then cross-reactivity due to sensitization to AK is still possible, however only Region 1 would be responsible for crustacean-cephalopod cross-reactivity.

## Discussion

The development of safe and reliable diagnostic tools is crucial to accurately diagnose allergic sensitization in patients and determine the primary allergen sources. Diagnosis of shellfish allergy, in particular, is a major challenge for the management of the allergic patients due to highly cross-reactive nature of some shellfish allergens. This problem is clearly evident in a recent study by Pascal et al. (6) where 44% of their negative controls positively reacted with tropomyosin, resulting in an overall false-negative rate of 17% in their diagnosis. A preceding study demonstrated that IgE recognition of allergic patients toward identical and/or similar homologous peptides to the allergen epitopes are the basis of the molecular cross-reactivity (31). Thus, the present study was conducted to determine shrimp IgE-binding epitopes that could be used to predict cross-reactivity toward other invertebrate species in shrimp allergic individuals, through which better predictive diagnostic tools for shellfish allergic patients could be developed.

The present study demonstrates that within a large directory of shrimp IgE-binding epitopes, there are a substantial fraction of epitopes that are highly conserved across invertebrate species. These conserved epitopes might play a key role in cross-reactivity between shrimp and other invertebrate species. Shrimp TM and AK shared a higher number of conserved epitopes compared to shrimp SCP and MLC; in fact, no conserved epitope could be found for SCP, while the MLC only shared two epitopes in one region with cockroach MLC. A recent study demonstrated the absence of IgE recognition to shrimp SCP and MLC in house dust mite allergic infants (7). These results suggest that TM and AK are the major contributing proteins in immunological and clinical cross-reactivity between crustacean and other invertebrate groups. Furthermore, comparative evaluation of the number of conserved epitopes in TM and AK revealed a clear cross-reactive hierarchy where cockroach is on the top followed by mite, while molluscs are on the bottom of this hierarchy.

House dust mite and cockroach are the most common sources of indoor allergens worldwide with up to 85 and 60% of asthmatic patients eliciting sensitization to at least one of the mite or cockroach allergens, respectively. In a retrospective study of 95 shrimp-allergic patients in Canada, 90.5% of those patients had a positive test to HDM (47). Similarly, a study on an Asian population found a majority of patients with shrimp allergy have positive skin tests to HDM and cockroach (48). Vivid evidence of this cross-reactivity was demonstrated by a study on Orthodox Jews who positively tested against shrimp yet had no prior exposure to seafood due to strict religious laws prohibiting shellfish consumption (14). TM and AK seem to be the allergens responsible for this cross-reactivity as supported by the current study. Shrimp TM shares about 82 and 81% amino acid identity with cockroach and house dust mite tropomyosin, respectively. Likewise, shrimp AK shares about 82 and 78% amino acid identity with cockroach and house dust mite AK, with 66% of shrimp TM IgE-binding epitopes and 80% of shrimp AK IgE-binding epitopes are also existing within TM and AK of cockroach and house dust mite. These identified IgE-binding epitopes could be used to diagnose shrimp sensitization in patients sensitized to cockroach or house dust mite, without a previous history of allergic reactions to shrimp. Reciprocally, we can use the non-conserved IgE-binding epitopes to diagnose shrimp mono-sensitization. These findings are of significant impact on the diagnosis of shrimp allergy as current diagnostic tools using tropomyosin are not specific. Although tropomyosin is a good predictor of allergy reaction to crustacean (49), the rate of false positive reaction is still high due to IgE binding of antibodies developed in patients against tropomyosin from other invertebrate sources, in particular house dust mite and cockroach (11). Furthermore, the identification of specific IgE binding epitopes allows the prediction of allergic reactions to ingested crustacean, in patients allergic to cockroach and house dust mite. As over 30% of the global population is sensitized to dust mite allergens, the developed predictive model in this study could be of major importance.

Crustaceans and molluscs are generally referred to as “shellfish” in the context of seafood consumption and avoidance to both groups are often advised for shellfish allergic patients (50). Patients with allergy to shellfish may fail to identify the offending seafood species, often as a result of confusion regarding the different common names used to describe diverse seafood. Crustaceans are classified as arthropods together with spiders and insects, while the group of molluscs is a large and diverse group, subdivided into different classes such as bivalves, gastropods, and cephalopods. Precise diagnosis of allergy to crustacean or mollusc species is difficult as no species-specific allergens have been identified so far. Moreover, true sensitization to shellfish-specific allergens can be hampered due to the highly cross-reactive nature of some allergenic proteins, including TM and AK (51, 52). While crustacean TMs and AKs show very high amino acid sequence identity (up to 98 and 97%, respectively) with demonstrated IgE cross-reactivity (53, 54), the reported sequence identity between crustacean and mollusc TMs and AKs is much lower with only up to 68 and 58%, respectively. The gold standard to determine food allergy is an oral food challenge. However, due to the risk of severe reactions to shellfish allergens, this test is not frequently performed. Our present findings suggest that specific shrimp allergen IgE-binding epitopes could be used as a robust, alternative way to diagnose cross-reactivity between crustacean and mollusc species among shellfish-allergic patients. Among the known 97 shrimp TM IgE-binding epitopes, 71 epitopes are only existing within crustacean TMs, while 26 epitopes are shared with mollusc TMs. Meanwhile, of the 39 shrimp AK IgE-binding epitopes, only five epitopes are shared with cephalopod AK. In contrast, no shrimp SCBP and MLC IgE-binding epitopes are present in mollusc SCBP or MLC. These findings indicate that only shellfish-allergic patients sensitized to TM have a risk of reacting to both crustacean and mollusc species. This supports the conclusion of the previous study where crustacean-allergic patients with concurrent mollusc allergy reacted more frequently to tropomyosin than without mollusc allergy (93 vs. 35%, respectively, *P* = 0.004), while recognition of the other allergens were not different in both patient cohorts (55).

Our findings also demonstrate different patterns of conserved IgE-binding epitopes among the three mollusc classes, suggesting that some crustacean-allergic patients will cross-react to one but tolerate another class of molluscs. The cephalopods have a higher probability to cross-react with crustacean than the other mollusc classes. The cephalopod TM amino acid sequences have a higher identity with crustaceans than those of gastropod and bivalve (68 vs. 63 vs. 62.3%, respectively), and therefore contained more homologous peptides of shrimp TM and AK IgE-binding epitopes. From the study of Vidal et al. (55), of the 14 crustacean-allergic patients with mollusc allergy that were examined by skin prick tests against different mollusc species, 11 patients were positive to cephalopods, and 6 patients were positive to bivalves. While no study identified IgE-cross-reactivity due to AK between crustacean and gastropods or bivalves, cross-reactivity between cephalopods and crustacean has been reported (56). Nevertheless, immunological cross-reactivity between shrimp and other mollusc classes, the gastropods and bivalves, has been demonstrated in several studies (57–59).

Gastropod and bivalve TMs share only 60% sequence identities with crustacean TMs, however, unlike cephalopod where the identity of TM amino acid sequence is very high among the group, the identity of TM amino acid sequence in the species among those two classes is very variable, particularly among bivalve species. The variability of bivalve TM is very apparent where out of 23 conserved IgE-binding epitopes, only five epitopes were shared across all species of bivalve providing a molecular basis of selective cross-reactivity (46). It is to be noted that most experimental IgE epitope mapping studies published in the database use short overlapping peptide libraries for each allergen. This inevitably leads to elucidation of only linear IgE epitopes. However, since tropomyosin has an alpha-helical structure, all its IgE binding epitopes are known to be linear epitope (29). In case of arginine kinase, there may be a possibility of conformational IgE epitopes but this hasn't yet been proven experimentally, and is a caveat of this study.

Based on the abovementioned findings on the different pattern of IgE-binding epitope conservations in the three mollusc classes, we developed a decision tree to predict immunological cross-reactivity between shrimp and mollusc classes based on TM and AK. This decision tree could contribute significantly toward patient management, particularly on the aspect of food avoidance and diet. It has been well-known that shrimp allergic patients are advised to avoid all shellfish species including mollusc due to the risk of cross-reactivity. Our decision tree suggests that this advice should not be generalized as only patients sensitized to TM which account for 70–80% of total shrimp-allergic patients have a risk of cross-reacting with mollusc allergens. Moreover, the cohort of shrimp-allergic patients could be further divided into five groups based on their cross-reactivity patterns to specific IgE binding epitopes.

## Conclusion

Prediction of immunological cross-reactivity between an allergen and close related proteins based on similarity of the IgE-binding epitopes has been confirmed to be more accurate than the prediction based on similarity of the complete amino acid sequence of the allergenic proteins. Food allergens including shellfish allergens tend to have sequential IgE-binding epitopes due to digestion in the gastrointestinal tract. Thus, epitope sequence comparison is more relevant and conceivable for assessing the potential cross-reactivity of allergenic proteins, then comparing the whole protein sequence. The shrimp allergen IgE-binding epitope conservation results outlined in this study illustrate that a clear hierarchy of cross-reactivity is discovered, with TM being the most cross-reactive allergen among allergenic invertebrate species. This is most likely the main reasons that TM is one, if not the major pan-allergen in inhalant and ingestion animal allergy. The IgE binding epitopes located at the N- and C-terminal regions of TM are highly conserved and could be used as biomarkers to predict allergic cross-reactivity of shrimp-allergic patients. Unexpectedly, more than half of the TM as well as the AK IgE epitopes were found to be conserved in cockroach and mite TM and AK, respectively. In contrast, only few shrimp IgE-binding epitopes were conserved across the molluscs. This suggests a low risk of cross-reactivity of shrimp allergic patients to molluscs, while a high risk of cross-reactivity to cockroach or mite is predicted. We developed for the first time a decision tree to predict cross-reactivity between shrimp and molluscs based on the major allergens TM and AK. These fundamental findings could simplify the diagnosis of cross-reactivity among shellfish-allergic patients, thereby avoiding potential life-threatening food challenges.

## Data Availability Statement

Publicly available datasets were analyzed in this study. This data can be found here: https://www.iedb.org/ - list of epitopes can be found in the Supplementary Material.

## Author Contributions

RN, SDK, and AL conceived the study and wrote the manuscript. RN, EJ, TR, and SK performed and analyzed the experiments.

### Conflict of Interest

The authors declare that the research was conducted in the absence of any commercial or financial relationships that could be construed as a potential conflict of interest.

## Supplementary Material

Data Sheet 1 List of epitopes.
